# Structural equation modeling of the quality of life for patients with marfan syndrome

**DOI:** 10.1186/s12955-016-0488-5

**Published:** 2016-06-02

**Authors:** Ju Ryoung Moon, Yong Ae Cho, June Huh, I-Seok Kang, Duk-Kyung Kim

**Affiliations:** Department of nursing, Grown-Up Congenital Heart Clinic, Heart Vascular and Stroke Institute, Samsung Medical Center, Seoul, Korea; Redcloss College of Nursing, Chung-Ang University, Seoul, Korea; Department of Pediatrics, Grown-Up Congenital Heart Clinic, Heart Vascular and Stroke Institute, Samsung Medical Center, Sungkyunkwan University School of Medicine, 81 Irwon-ro, Gangnam-gu, Seoul, 135-710 Korea; Department of Medicine, Division of Cardiology, Heart Vascular and Stroke Institute, Samsung Medical Center, Sungkyunkwan University School of Medicine, Seoul, Korea

## Abstract

**Background:**

We used structural equation modeling to evaluate the quality of life (QOL) for patients with Marfan syndrome (MFS). The goal was to provide guidelines to facilitate the development of interventions and strategies to improve the QOL for patients with MFS.

**Methods:**

The participants fulfilled the Ghent 2 criteria for MFS and they comprised patients who visited the cardiology outpatient department of a tertiary hospital in Seoul, Korea, between August 17, 2013 and April 17, 2014. Demographic, social support, disease-related factors, biobehavioral factors, and QOL data were collected in one-on-one interviews.

**Results:**

The final analyses included 218 patients. Anxious and depressed patients comprised 63.8 and 71.5 % of the sample, respectively. For the hypothetical model, the goodness-of-fit index = 0.91, normal fit index = 0.93, and comparative fit index = 0.90. The outcome was suitable for the recommended level, so the hypothetical model appeared to fit the data. In patients with MFS, the QOL was affected significantly by social support, disease-related factors, and biobehavioral factors. These variables explained 72.4 % of the QOL in patients with MFS. Biobehavioral factors had the strongest and most direct effects on QOL.

**Conclusion:**

To improve QOL in patients with MFS, comprehensive interventions are necessary to assess and manage biobehavioral factors, social support, and disease-related factors.

## Background

Marfan syndrome (MFS) is a genetic disease caused by a mutation in the fibrillin-1 gene, which controls a component of connective tissue [1]. The average life expectancy of individuals with MFS has been extended and it is similar to that of healthy people when patients receive appropriate interventions, such as the administration of beta-blockers, restrictions on physical activity, and aortic surgery [2].

These medical treatments have improved the survival rate and health status of patients with MFS [3]. However, patients with MFS are still susceptible to sudden death with aortic dissection or rupture, which may occur at any time in their lives [1]. In addition, patients with MFS may experience the burden of numerous instances of vascular surgery, the administration of medication throughout the lives, restricted physical activity, pain, and chronic fatigue [3–5]. There is a >50 % possibility of the disease being transmitted to the children of patients with MFS [6] and they have distinct physical characteristics [7, 8]. All of these issues result in emotional distress in patients with MFS, including anxiety and depression [3–9]. Most patients with MFS suffer from physical and psychological issues throughout their lives [4, 10]. Therefore, it is necessary to consider physical and psychological aspects when assessing the overall quality of life (QOL) in patients with MFS.

According to previous studies, the main factors that influence that QOL in patients with MFS comprise MFS-related physical symptoms, anxiety, depression, and social support [3–12]. However, VanToerloo and De Paepe found that the incidence of depression and anxiety by patients with MFS did not differ significantly from that in the normal population [10]. In addition, most previous studies investigated the impacts of single factors on the QOL of individuals with MFS, but various factors can affect the QOL in multifaceted ways, both directly and indirectly. Previous studies have reported that demographic factors [5, 9, 13] and disease-related physical symptoms [3, 5, 8, 11, 12] (e.g., aortic proximal dilatation and clinical symptoms) influence the QOL, but they also affect the prevalence of QOL-related factors such as depression and fatigue [5, 14, 15]. Moreover, these studies found that pain [3, 14], fatigue [3, 5, 14, 15], and body image [3, 7] were related to the QOL of individuals with MFS, as well as the variables that influence depression and anxiety. These biobehavioral factors, including anxiety, depression, fatigue, pain, and body image, combined with social support will have complex effects on the QOL of patients with MFS [4, 16, 17].

However, no previous studies have constructed or verified a comprehensive structural model of the relationships among the various factors that may affect the QOL of patients with MFS, including biobehavioral factors, to identify the direct or indirect relationships among these factors. In particular, there is a need for a QOL model of patients with MFS in Korea because the social, cultural, and physical characteristics of these patients may differ from those in other countries, as described in previous studies. In addition, structural model validation is required to establish a strategic plan for improving the QOL of patients with MFS.

### Purpose

The aim of this study was to build a QOL structural model of patients with MFS, verify its goodness of fit, and determine the factors that affect the QOL, as well as their direct or indirect relationships. After detect the QOL status, they may be considered about this problems and advice to helping about their specific issues.

## Conceptual framework and hypothetical research model

**Fig. 1:**
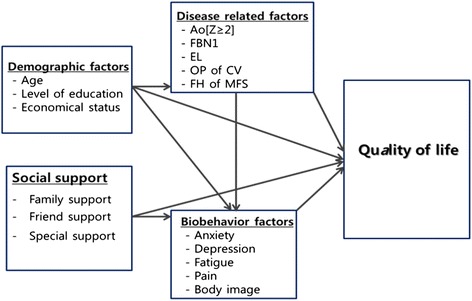
Research framework. FH of MFS = family history of Marfan syndrome; Ao = sinus of Valsalva of diameter Z (= Z score), indicating the presence of aortic root dilatation (when standardized with respect to age and body size); FEN 1 = fibrillin-1 mutation; EL = ectopia lentis; OP of CV = operation on the cardiovascular system

Based on a literature review and previous studies, we determined that demographic factors, social support, disease-related factors, and biobehavioral factors affect the QOL of patients with MFS directly or indirectly. Figure 1 show the conceptual framework employed in this study.

## Methods

### Research design

We developed an exploratory structural model study to identify the factors that affect the QOL of patients with MFS. We then examined the direct and indirect relationships among these factors.

### Research subjects

The inclusion criteria comprised adult patients aged ≥20 years who were diagnosed with MFS based on the revised Ghent guidelines [1]. The exclusion criteria comprised patients with a history of psychiatric disorder, such as schizophrenia or bipolar disorder, and organic psychotic symptoms, or who had taken prescribed psychotic drugs, such as antidepressants, for more than two weeks. The study period ranged from August 17, 2013 to April 20, 2014. In total, 239 patients visited the Samsung Medical Center MFS Clinic during this period. We excluded 21 patients, i.e., 16 because they had taken antidepressants for more than two weeks or they had been diagnosed with schizophrenia or bipolar disorder, and five because they responded inadequately to the survey questions. Thus, the final analysis included 218 patients. The sample size satisfied the requirements for structural equation modeling analysis (i.e., a sample size ≥200) [18, 19].

### Instruments

#### QOL

QOL was measured with the Korean version of the 36-item Short-Form Health Survey (SF-36), which was developed by Ware and Sherboune [20, 21]. It was translated into the Korean version and tested by Nam and Lee [22]. The SF-36 questionnaire was designed to measure eight health concepts: limitations on physical activities and the usual roles of activities due to physical health problems; limitations on social activities because of physical or emotional problems; general mental health (psychological distress and well-being); bodily pain; limitations on the usual roles of activities due to emotional problems; vitality (energy and fatigue); and general health perception. The items from each concept were summed and rescaled over a range of 0–100, where 100 represented the best health-related QOL. The scores on the subscales were aggregated into two standardized summary scores: physical component summary (PCS) and mental component summary (MCS) [20, 21]. Quality Metric Health Outcome Scoring Software 4.5 was used to calculate the QOL scores in the present study [23]. The Korean version of SF-36 has adequate internal consistency (0.92–0.94), test/retest reliability (0.71–0.89), and construct validity [24]. In this study, Cronbach’s α for the SF-36 was 0.89 based on the total score, with 0.88 for PCS and 0.91 for MCS.

#### Social support

Social support was evaluated using the Multidimensional Scale of Perceived Social Support (MSPSS) tool, which was developed by Zimet et al. [25] and a version was translated into Korean by Shin and Lee [26]. The MSPSS tool comprises 12 items and uses a five-point scale to assess family support, friend support, and special support. The possible score ranges among 12–60 points, where higher scores represent better social support. Cronbach’s α for the reliability of the original tool was 0.83 [25] and 0.89 in the present study. Cronbach’s α for family support, friend support, and special support were 0.93, 0.87, and 0.89, respectively.

#### Disease-related factors

Disease-related factors are components of the revised Ghent nosology [1]. They comprise the diameter of the sinus of Valsalva according to echocardiography, the presence or absence of the fibrillin-1 mutation based on genetic analysis, intraocular lens dislocation, the number of thoracic and abdominal aortic surgeries, and the presence/absence of a family history of MFS.

#### Biobehavioral factors

Biobehavioral factors are personal responses to a disease, which include emotional and physiological processes [27, 28]. In this study, these factors comprised depression, anxiety, fatigue, pain, and body image. These biobehavioral factors were identified based on previous studies, which demonstrated that pain [3, 14], fatigue [3, 5, 14, 15], body image [3, 7], and anxiety and depression have significant relationships with each other [27, 28].

1. Anxiety and depression: Anxiety and depression were measured with the Hospital Anxiety Depression scale of Korea (HAD-K), which was developed by Zigmond and Snaith [29] and translated into Korean by Oh et al. [30] The HAD-K comprises 14 questions, where even numbers are questions related to depression and odd numbers address anxiety. Each question is assessed on a four-point scale, where a total score of <8 points denotes no depression/anxiety, 8–10 points denote borderline depression/anxiety, and >11 points signifies clinical depression/anxiety [29]. For the original tool, Cronbach’s α was 0.89 for depression and 0.79 for anxiety [29], whereas in this study, the values were 0.82 for depression and 0.85 for anxiety.
2. Fatigue: Fatigue was measured with the Fatigue Severity Scale, which was developed by Krupp et al. [31] and translated into Korean by Kim [27]. The possible scores range among 9–63 where a higher score indicates a more severe degree of fatigue.
3. Pain: We used a 10-cm visual analog scale (VAS) to assess pain. The left-hand side of the VAS was recorded as no pain whereas the most severe pain was recorded at the end of the right-hand side. Chest pain, back pain, and muscle pain were assessed and recorded during the previous four weeks.
4. Body image: Body image was measured using the Body Image States Scale (BISS) developed by Cash et al. [32]. Permission was obtained from the authors to translate the BISS into a Korean version for this study. The translation was processed according to Brislin’s translation model [33]. The BISS comprises six questions about physical appearance and it utilizes a nine-point scale. Reverse scoring was used to score the even numbered questions. The total possible score ranged among 6–63 points where a higher score denoted a more positive body image. Cronbach’s α for the original tool was 0.85 [32] and it was 0.83 in the present study.

### Data collection

To protect the subjects, the survey was conducted after obtaining approval (no. 2013–08–016) from the Institutional Review Board of the Samsung Medical Center. If the subjects agreed to participate in the study, they were asked to sign a consent form and to complete a questionnaire. One researcher and a cardiovascular center outpatient nurse who served as a research assistant collected the survey data during one-on-one interviews when the patient visited the outpatient clinic for check-ups or tests. Before collecting the data, the chief of research met with the research assistant three times to discuss the purpose and risks of the study, ethical aspects related to patients, and the survey tools used for data collection. To ensure the consistency of the research methods between interviewers, the interviews were performed together for the first five patients before subsequent data collection. After the data collection process commenced, the researcher and the research assistant had a consultation meeting each week to discuss any issues that emerged during the interviews. The researcher reviewed the medical records and collected the patient’s clinical information.

### Statistical analysis

The data were analyzed using SPSS (v. 21.0) and AMOS (v. 21.0) software. Descriptive statistics were used to analyze demographic factors, social support, physical factors, biobehavioral factors, and the QOL of patients with MFS. Pearson’s correlation coefficient was used to determine the multicollinearity between the variables. The generalized least squares method was used because the model satisfied normality for kurtosis and skewness, but it did not satisfy multivariate normality. The following were used in the goodness-of-fit tests for the model: *χ*^2^, degrees of freedom (df), goodness-of-fit index (GFI), normal fit index (NFI), comparative fit index (CFI), root mean squared error of approximation (RMSEA), Tucker–Lewis index (TLI), and the parsimonious goodness-of-fit index (PGFI).

## Results

### Subject characteristics

In total, 137 (62.8 %) of the patients were men and the mean age was 36.3 ± 4.5 years. Among the patients, 166 (76.2) were college graduates, 150 (69.2) were employed, and 145 (66.5 %) were married. The mean height of the patients was 178.4 ± 12.5 cm. After standardizing for age and weight, 167 (76.6) patients had an abnormally dilated aorta and 99 (45.2 %) patients possessed the fibrillin-1 mutation according to genetic tests. In addition, 163 (74.8) patients had past medical history of more than one cardiovascular surgery and 79 (36.2 %) had a family history of MFS (Table 1).

**Table 1:** Demographic & clinical characteristics of subjects (*N* = 218)

Characteristics	Categories	N (%) or Mean ± SD	Range
Gender	Male	137 (62.8)	
	Female	81 (37.2)	
Age (year)		36.3 ± 4.5	18–62
	Male	37.5 ± 5.7	18–57
	Female	35.1 ± 3.4	20–62
Education level	High school	52 (23.8)	
	≥ College	166 (76.2)	
Occupation	Yes	150 (69.2)	
Marital status	Single	49 (22.4)	
	Married	145 (66.5)	
	Divorced/Widowed	24 (11.1)	
Monthly expenditure (10,000 won)	< 120	38 (17.5)	
	120–319	114 (52.3)	
	≥ 320	66 (30.2)	
Height(cm)		178.4 ± 12.5	156.3–192.6
Ao.(Z > 2)^a^	Yes	167 (76.6)	
Fibrillin-1 mutation (via gene study)	Yes	82 (37.6)	
Operation for cardiovascular system (frequency)	0	55 (25.2)	
	1	111(50.9)	
	2	40 (18.3)	
	3	12 (5.6)	
Family history of MFS^b^	Yes	79 (36.2)	

The values are expressed as mean ± standard deviation; and qualitative variables, as percentages of the total. ^a^ *Ao* sinus of Valsalva of diameter, *Z* Z score, the presence of aortic root dilatation (when standardized to age and body size); MFS^b^ = Marfan syndrome

### Descriptive statistics

The mean, standard deviation, and ranges of the variables used in this research model are shown in Table 2. The kurtosis and skewness values for all of the variables used in this study were less than ±1.96 (Table 2) and the assumption of a normal distribution was satisfied [10, 12].

**Table 2:** Descriptive statistics and test for normality of observed variables (*N* = 218)

Variables	N (%) or Mean ± SD	Range	Skewness	Kurtosis
Social support	41.3 ± 8.5	19.1–55.6	-.11	.07
Anxiety	18.5 ± 4.3	7.0–26	-.15	−1.30
No	13 (5.9)			
Borderline	66 (30.3)			
Yes	139 (63.8)			
Depression	16.0 ± 3.9	7.0–26	-.08	−1.22
No	4 (1.8)			
Borderline	58 (26.7)			
Yes	156 (71.5)			
Fatigue	46.3 ± 6.5	30.2–59.1	.13	.28
Pain	6.9 ± 2.3	4.2–8.5	1.04	-.72
Body image	15.2 ± 5.6	9.6–25.1	-.01	-.75
Quality of life	42.8 ± 10.3	22.2–86.2	.18	.30
Multivariate				18.96

The values are expressed as mean ± standard deviation; and qualitative variables, as percentages of the total

### Correlation and multicollinearity analysis of the variables

Before hypothesis testing, we conducted correlation analysis using the measured variables. Lower QOL was associated with older age (r = −0.25, *P* = 0.013), lower educational level (r = 0.42, *P* = 0.012), lower economic status (r = −0.15, *P* = 0.024), lower social support (r = 0.49, *P* < 0.001), increased number of cardiovascular surgeries (r = −0.56, *P* < 0.001), increased anxiety (r = −0.59, *P* < 0.001), increased depression (r = −0.67, *P* < 0.001), greater fatigue (r = −0.52, *P* < 0.001), higher pain scores (r = −0.64, *P* < 0.001), and lower body image (r = 0.50, *P* < 0.001). The absolute values of the correlation coefficients determined between the pairs of independent variables were all <0.70. Therefore, multicollinearity was not present in the data [18, 19].

### Testing the structural model of the QOL of patients with MFS

#### Feasibility assessment for the hypothetical model

We conducted a confirmatory factor analysis of the measurement model in step 1. The confirmatory factor analysis was performed with demographic factors, social support, disease-related factors, biobehavioral factors, and QOL, whereas we excluded single measurement latent variables. Based on the disease-related factors, the factor loading for crystalline lens dislocation was 0.09, which was below the reference value range of 0.5–0.95. Thus, this factor was removed because of its poor fit with the measurement model [18, 19].

#### Test of the goodness of fit of the hypothetical model

The results of the analysis of the structural equation model produced using the study variables in the hypothetical model were as follows: goodness of fit for *χ*^2^ = 151.30 (*P* < 0.001, df = 45), GFI = 0.91, RMSEA = 0.05, NFI = 0.93, CFI = 0.92, TLI = 0.97, PGFNI = 0.46, and PNFI = 0.44. All of the GFI indices satisfied the recommended levels.

#### Analysis of the hypothetical model

**Fig. 2:**
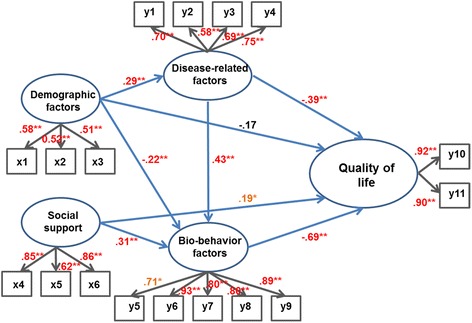
Path diagram for the hypothetical model. ^*^ *P* < 0.05; ^**^ *P* < 0.01. x1 = Age; *x*2 = Education level; x3 = Economic status; x4 = Family support; x5 = Friend support; x6 = Special support; y1 = Dilatation of sinus of Valsalva; y2 = Fibrillin-1 mutation; y3 = Family history of Marfan syndrome; y4 = Frequency of operations on the cardiovascular system; y5 = Anxiety; y6 = Depression; y7 = Fatigue; y8 = Pain; y9 = Body image; y10 = Physical component score; y11 = Mental component score

The results of the analysis of the hypothetical model are as follows (Fig. 2). In the hypothetical model, the following were statistically significant: disease-related factor path in the demographic factors (*P* < 0.001), biobehavioral factor path in the demographic factors (*P* < 0.001), biobehavioral factor path in the social support path (*P* < 0.001), QOL path in the social support path (*P* < 0.05), QOL path in the disease-related factor path (*P* < 0.001), biobehavioral factor path in the disease-related factor path (*P* < 0.001), and QOL path in the biobehavioral factor path (*P* < 0.001). However, the QOL path (*P* = 0.432) was not statistically significant in the demographic factors. The modification indices for the other paths were all <10.0 and none of the paths required further analysis.

#### Effectiveness analysis of the hypothetical model

The direct, indirect, and total effects of the factors associated with the QOL of the patients with MFS are presented in Table 3. The biobehavioral factors had the greatest direct effect on the QOL with a score of 0.695. The disease-related factors had a direct effect on the QOL with a path coefficient of 0.391, and a total effect of −0.091 when added to the indirect effect of the biobehavioral factors (0.300). Social support had a total effect of 0.172 on the QOL. Social support, disease-related factors, and biobehavioral factors explained 72.4 % of the QOL of the patients with MFS. Demographic factors, social support, and disease-related factors explained 52.2 % of the QOL. Demographic factors also explained 12.4 % of the disease-related factors.

**Table 3:** Standard direct, indirect and total effect

Endogenous variables	Exogenous variables	Standardized direct effect	Standardized indirect effect	Standardized total effect	SMC
Quality of life	Demographic factors	-.172	-.182	.354	.724
	Social support	.193*	.173	.366	
	Disease-related factors	-.391**	-.196	.587	
	Biobehavior factors	-.695**		-.695	
Bio-behavior factors	Demographic factors	-.220**	-.203	-.423	.522
	Social support	.313**		.313	
	Disease-related factors	.433**		.433	
Disease-related factors	Demographic factors	-.221**		-.221	.124

*SMC* Squared multiple correlations

*P < .05; **P < .01

## Discussion

In this study, we aimed to construct a hypothetical model and verify the significance of the direct/indirect paths and the goodness of fit of the model under the theoretical assumption that demographic factors, social support, disease-related factors, and biobehavioral factors, including depression, anxiety, fatigue, pain, and body image, determine the QOL of patients with MFS directly and indirectly. This study is significant because it is the first analysis of the QOL of patients with MFS in Korea.

According to this structural model, social support, disease-related factors, and biobehavioral factors explained 72.4 % of the QOL for MFS subjects. Direct comparisons with the findings of other studies are difficult because there are no other comprehensive QOL models of patients with MFS, or alternative hereditary diseases, from Korea or other countries. However, although the patient group was different, a structural model that targeted patients with osteoarthritis [34] had explanatory power of more than 63.6 %. This difference may be attributed to the inclusion of biobehavioral-related factors in the present study, whereas the other study focused only on the physical and psychological adaptation of patients with degenerative arthritis. Studies of Korean stroke patients [35] and chronic kidney failure patients [27] have found that depression, anxiety, fatigue, and pain affect the QOL of patients, thereby demonstrating that biobehavioral factors have a significant impact on QOL in patients. In previous studies, anxiety and depression were the most important biobehavioral factors in patients with MFS [3–7].

Studies have shown that pain [3, 14] caused by dural ectasia and surgery, fatigue [3, 5, 14, 15], and body image issues [3, 7], such as great height, long and thin fingers, scoliosis, and the need for thick eyeglasses, were associated with depression, anxiety, and QOL. Depression, anxiety, pain, fatigue, and the body image of patients with MFS could influence the QOL either independently or in complex combinations. Previous studies have shown that each of these variables affects the QOL of patients with MFS independently [4, 9], but no studies have examined the comprehensive effects of all of these variables on the QOL. In this study, we defined depression, anxiety, fatigue, pain, and body image as biobehavioral factor variables that affect patients with MFS, and we analyzed the paths and the degrees of these factors with respect to QOL in patients.

Confirmatory factor analysis of each of the biobehavioral factors showed that all the variables had a loading of >0.70, which indicated that it was reasonable to group them into biobehavioral factors. According to the results of this study where we defined depression as a biobehavioral factor, 98.2 % of the patients were found to have depression, including borderline depression, which demonstrates that most patients with MFS experienced depression. These results are partly consistent with those reported by Fusar-Poli et al. [9] who found that depression and schizophrenia were prevalent among patients with MFS due to the possibility of sudden death caused by aortic rupture, in addition to limitations in terms of physical activity and exercise, the need for lifelong medication, and a high risk of second-generation heritability. However, this was a high rate of experience of depression compared with the results reported by Peter et al. [4] who found that only 46 % experienced depression using the Center for Epidemiological Studies Depression Scale. Moreover, the study by Peter et al. [4] used different tools so it was difficult to compare their results with those obtained in the present study, which may also be attributable to differences among the participants. In particular, the participants in the present study were fairly young and they comprised a higher number of males than females, where most had a high education level. These factors may have affected the reported experience of depression. This is partially supported by the findings of Fusar-Poli et al. [9] who reported that older patients and male patients had poor mental well-being. The results of the latter study showed that 93 % of the patients had both depression and anxiety; indeed, anxiety and depression are strongly related. In addition, age, educational level, economic condition, social support, number of surgeries, and the presence/absence of a family history of the disease were associated with anxiety and depression according to the additional analysis performed in this study. Depression and anxiety are significantly associated with perceived stigma [7] and coping strategies [7, 9], but this area still requires further research.

Furthermore, the analysis of our structural model showed that biobehavioral factors had direct effects on the QOL, but there were also important roles for demographic characteristics, social support, and disease-related factors. Therefore, the results of this study demonstrate that multifaceted elements, including biobehavioral factors, are important variables for explaining the QOL of patients with MFS. Moreover, this study highlights the importance of biobehavioral factors and the need for biobehavioral interventions to address clinical care issues [30].

Among the variables, we found that disease-related factors had the greatest impact on biobehavioral factors. We selected the main disease-related factors based on the guidelines in the Ghent criteria, which are used to diagnose MFS. The Ghent criteria comprise aortic dilatation, the presence of a mutation in the fibrillin-1 gene according to genetic tests, and the presence/absence of a family history of the disease [1]. The number of surgeries was added to these factors in our study. Our findings confirm that QOL, in addition to depression and fatigue [3, 5], is related to aortic proximal dilatation and a definite diagnosis by genetic testing [36, 37]. Biobehavioral changes are likely to occur in patients with gradually progressive aortic dilatation who have been diagnosed by genetic testing and who have undergone multiple cardiovascular operations. These patients require special attention and care.

The results of this study also demonstrate that social support influenced biobehavioral factors. When we analyzed the association between social support, biobehavioral factors, and depression, we found that social support had a significant influence on depression. These results are consistent with those obtained by Cohen and Biesecker who described the role of social support in depression [16]. In addition, we separately analyzed the level of support perceived by the patient, which showed that support from the nurse, spouse, and family were the only support factors that decreased depression in patients. This is mainly attributable to the cultural characteristics of Korea, which places a great emphasis on blood ties.

Based on these results, approaches should be developed for effectively managing biobehavioral factors, including anxiety, depression, fatigue, pain, and body image, to improve the QOL of patients with MFS. These approaches could enhance the QOL because biobehavioral factors may be adjusted to manage patients by considering the progression of aortic dilation, the identification of MFS genes, the number of cardiovascular surgeries, and the presence or absence of a family history as disease-related factors.

Thus, QOL may be improved by managing biobehavioral factors, which are influenced by disease-related factors, the progression of aortic dilation, and the identification of MFS genes, the number of cardiovascular surgeries, and the presence or absence of a family history. Developing and providing intervention programs to enhance social support may reduce biobehavioral changes, such as depression, which may be a good strategy for improving the QOL of patients with MFS.

Our investigation differs from previous studies because we considered the QOL of Korean patients with MFS for the first time. Furthermore, this is the first study in Korea or other countries to show that multiple variables (i.e., social support, disease-related factors, and biobehavioral factors) can affect the QOL of patients with MFS.

A limitation is that this was a single study where 62.8 % of the patients were male, relatively young, and highly educated. The participants were patients with mild MFS who could visit outpatient clinics and those with severe depression who had difficulty visiting outpatient clinics were not included. Thus, the results of the study must be generalized with care. In addition, we did not use a disease-specific QOL tool that was developed for patients with MFS. The reliability and validity of the tool that we employed was verified previously in a healthy population and it is applied widely to chronic disease patients rather than those specifically with MFS. The reliability of this tool was satisfactory in the present study, but we suggest that follow-up studies should be performed to develop and apply a disease-specific QOL tool for patients with MFS.

## Conclusion

In this study, we analyzed the factors that affect the QOL of patients with MFS and we constructed a model to identify direct and indirect paths. All of the GFI indices satisfied the recommended levels.

According to this structural model, social support, disease-related factors, and biobehavioral factors explained 72.4 % of the QOL for MFS subjects. Biobehavioral factors explained 39.2 % of the social support and disease-related factors. In addition, demographical factors explained 12.4 % of the disease-related factors.

Based on these results, approaches should be developed for effectively managing biobehavioral factors to improve the QOL of patients with MFS. These approaches could enhance the QOL because biobehavioral factors may be adjusted to manage patients by considering disease-related factors.

Developing and providing intervention programs to enhance social support may reduce biobehavioral changes, such as depression, which may be a good strategy for improving the QOL of patients with MFS.
